# Psychobiotics and the need for better interventional data

**DOI:** 10.1186/s12916-023-03052-w

**Published:** 2023-09-01

**Authors:** BMC Medicine

**Affiliations:** The Campus, 4 Crinan Street, London, UK

Gut microbiota composition has been associated with multiple health outcomes: various studies employing predictive genetic studies and randomised controlled trials suggest this is the case. More specifically, it has long been postulated that the direct connections between the central nervous system and the gut may make the contents of the intestines a key regulator in a variety of central nervous system disorders. Probiotics comprise live bacteria and yeasts ingested as food supplements. Whilst the interest in and availability of probiotics for a range of disorders grows every year, the evidence to support such interventions is unclear. With the rise of “psychobiotics”, where does the clinical utility of interventions into gut microbiota as a treatment for neurological disorders currently sit?

Interventions targeting the gut microbiota including either probiotics (live bacteria or yeasts as food supplements) or FMT (faecal microbiota transplantation) seek to alter the composition of microbes in the individual’s intestinal tract. The downstream effects of this have been proposed to positively impact obesity, cystitis, blood pressure, susceptibility to infection, eczema, asthma and preeclampsia amongst other health outcomes. Amongst these, newer research points to a link between probiotics and mental health/neurological outcomes.

“Psychobiotics” is a new term coined to describe probiotic interventions which produce a health benefit in individuals with psychiatric or neurological disorders. The incidence of these disorders is on the rise globally and, following the global COVID-19 pandemic, is being diagnosed with greater frequency. Between 1990 and 2019, the disability-adjusted life-years attributed to mental disorders increased from 80.8 million to 125.3 million. As such, novel interventions which can prevent or modulate neurological disorders are of high interest. Indeed, the Future Market Insights estimates the current global market at $140.3 M with a projected worth of 201.8 M by 2033.

The concept of the gut-brain axis is decades old and is based around the direct neuronal connection between the two organs via the vagus nerve and the role of gut microbiota in production and modulation of neurotransmitters such as serotonin and dopamine. A vast amount of data has been published on this topic using different approaches such as animal model-based, observational, association, mendelian randomisation bioinformatic and human intervention studies. However, a surfeit of data from limited studies with conflicting or weak results will result in unclear clinical utility.

Amongst all mental health conditions studied, depression and anxiety symptoms have been identified in studies as being most meaningfully impacted by probiotic interventions. Since 2016, over a dozen trials have looked at the impact of probiotic supplementation over a period of 4–24 weeks on depression as ascertained by clinical score outcomes. Many of the trials have identified a positive impact of probiotic intervention particularly on individuals living with mild or moderate depression. Few of the studies included a robust follow-up period to ascertain longevity of effect, however.

Research has also been conducted on probiotics and FMT in animal models of Alzheimer’s disease (AD) looking at downstream effectors. Much of the research identifies markers such as APPα, AB40, AB42 and SOD levels and changes in these because of continuous probiotic exposure. These results indicate a potential role for probiotics; however, reliable research in humans with robust clinical endpoints is still needed to provide the basis for reliable interventions. Interestingly, a number of studies have found only moderate impacts of probiotic supplementation on cognitive function. In most cases, the trials are relatively small with short follow-up periods and with effects only present in individuals with mild cognitive impairment as opposed to Alzheimer’s disease. As such, the consensus on the utility of probiotics in Alzheimer’s is not clear. At present, the US Clinical Trials Registry lists a further two trials recruiting participants on the topic of Alzheimer’s disease and probiotics and only one of which includes clinical outcomes as its primary outcome.

Probiotic supplements are regulated under the umbrella of food in many regions and as such are not subject to drug regulations which validate efficacy. The issue of uncertainty around which specific bacteria provide lasting effective clinical outcomes can result in poorly efficacious products. Incorrect claims on the efficacy of psychobiotics may lead to adverse events such as digestive symptoms, headaches, allergy symptoms and increased infection risk whilst wasting money on a potentially ineffective intervention. The widespread commercial availability of these products, the potential adverse effects and economic negatives for the public means confusing poorly clinical research further muddies the issue. The US Clinical Trials Registry alone registers several trials in recruitment or active looking at the impact of probiotics or FMT on multiple sclerosis, epilepsy, migraine, schizophrenia and a further range of psychiatric/neurological-based disorders. What is clear is that in the field of psychobiotics, well-powered longitudinal trials with robust clinical outcomes are still needed. Specifically, any trials looking at psychobiotics must include robust standardised measures of disease progression and resolve issues of dosage and specific bacteria. Another approach, with the wide availability of probiotics, may be using real-world data. Strictly conducted large-sized studies focused on a single intervention with robust measurements can also provide useful clinical utility. Non-invasive, easy to administer, adverse event-free interventions for neurological disorders remain the holy grail. The current evidence suggests psychobiotics research still has some way to go before meeting this bar.

The gut microbiota presents a potentially potent target for modulation of the central nervous system. Improvements in the regulation and validation of products available to the public will help to improve both the effectiveness of interventions and avoid misuse or wastage. High-quality research will improve the development of more effective probiotics and is urgently needed.

For more discussion on interventions into neurological disorders, read our upcoming collection on dementia prevention and therapies.

## Data Availability

Not applicable.

